# Development, implementation, and evaluation of a curriculum for medical students on conflicts of interest and communicating risk

**DOI:** 10.3205/zma001296

**Published:** 2020-02-17

**Authors:** Nicole Deis, Cora Koch, Nadine Dreimüller, Eva Gaitzsch, Jens Weißkircher, Jana Jünger, Klaus Lieb

**Affiliations:** 1 Institute of Medical and Pharmaceutical Examination Questions, Mainz, Germany; 2 University Hospital of Mainz, Department of Psychiatry, Mainz, Germany; 3 University Hospital of Heidelberg, Chest Clinic, Heidelberg, Germany

**Keywords:** communication of risks, conflicts of interest, risk competence, curriculum development, shared decision-making

## Abstract

**Background: **Insufficient risk competence of physicians, conflicts of interests from interactions with pharmaceutical companies, and the often distorted presentation of benefits and risks of therapies compromise the advising of patients by physicians in the framework of shared decision-making. An important cause of this is that teaching on this subject is mostly lacking, or fragmented when it does take place [1], [2], [3], [4]. Even though the *German National Competence-Based Catalog of Learning Goals in Medicine* defines learning goals on the topics of conflicts of interest and communication of risk, there are no classes that integrate both topics. Our goal was to develop a model curriculum to teach conflicts of interest and communication of risk that would integrate statistical know-how, communicational competency on the presentation of benefits and risks, and the meaning and management of conflicts of interest.

**Project Description:** The development of the curriculum took place according to the six-step cycle of Kern et al [5]. An integrated curriculum was conceptualized, piloted, and adapted with the support of experts for the topics of shared decision-making, conflicts of interest, and communication of risk. The final version of the curriculum was implemented at the medical schools of Mainz and Heidelberg and evaluated by the students.

**Results: **The final curriculum consists of 19 lesson units. The contents are the fundamentals of statistics, theory of risk communication, practical exercises on communication of risk, and the fundamentals of the mechanisms of effect of conflicts of interest, recognition of distortions in data, and introductions to professional management of conflicts of interest. The course was implemented three times at two different medical schools with a total of 32 students, and it was positively rated by most of the 27 participating students who evaluated it on the 1-6 German school grading scale (mean: 1.4; SD: 0.49; range: 1-2).

**Discussion: **The curriculum we developed fills a gap in the current medical education. The innovative concept, which sensibly connects the transmission of theory and practice, was positively received by the students. The next steps are an evaluation of the curriculum by means of a two-center randomized study and the implementation at German and international medical schools. The process should be accompanied by continuous evaluation and further improvement.

## Zusammenfassung

**Hintergrund:** Mangelnde Risikokompetenz von Ärzt*innen, Interessenkonflikte aus Interaktionen mit pharmazeutischen Unternehmen und die häufig verzerrte Darstellung zu Nutzen und Risiken von Therapien beeinträchtigen die Beratung von Patient*innen durch Ärzt*innen im Rahmen der partizipativen Entscheidungsfindung. Eine wichtige Ursache ist die größtenteils fehlende und, wo vorhanden, nur fragmentierte Lehre zur Thematik [1], [2], [3], [4]. Trotz der im Nationalen kompetenzbasierten Lernzielkatalog Medizin (NKLM) in Deutschland definierten Lernziele zu den Themenbereichen Risikokommunikation und Interessenkonflikte fehlen Lehrveranstaltungen, die beide Bereiche integrieren.

Ziel war die Entwicklung eines Mustercurriculums für die Lehre von Risikokommunikation und Interessenkonflikten, das statistisches Know-how, Kommunikationskompetenz zur Darstellung von Nutzen und Risiken sowie die Bedeutung und den Umgang mit Interessenkonflikten vereint.

**Projektbeschreibung:** Die Entwicklung des Curriculums erfolgte nach dem 6-schrittigen Zyklus von Kern et al. [5]. Mit Unterstützung durch Experten für die Themenbereiche partizipative Entscheidungsfindung, Risikokommunikation und Interessenkonflikte wurde ein integriertes Curriculum konzipiert, pilotiert und adaptiert. Die Endversion des Curriculums wurde an denmedizinischen Fakultäten Mainz und Heidelberg implementiert und durch Studierende evaluiert.

**Ergebnisse: **Das endgültige Curriculum besteht aus 19 Unterrichtseinheiten. Inhalte sind Grundlagen der Statistik, Theorie der Risikokommunikation, praktische Übungen zur Risikokommunikation sowie Grundlagen zu den Wirkmechanismen von Interessenkonflikten, Erkennung von Verzerrungen in Daten und Anleitungen zum professionellen Umgang mit Interessenkonflikten. Es wurde drei Mal an zwei unterschiedlichen Fakultäten mit insgesamt 32 Studierenden implementiert und durch die teilnehmenden Studierenden überwiegend positiv evaluiert (Schulnote 1,4; N=27; SD=0,49; Range=1-2).

**Diskussion: **Das von uns entwickelte Curriculum schließt eine Lücke in der derzeitigen medizinischen Lehre. Das innovative Konzept, welches die Vermittlung von Theorie und Praxis sinnvoll verknüpft, wurde von den Studierenden positiv angenommen. Die nächsten Schritte sind eine Überprüfung der Wirksamkeit des Curriculums mittels einer bizentrischen randomisierten Studie sowie die Implementierung an deutschen und internationalen Fakultäten. Der Prozess sollte von einer kontinuierlichen Evaluation und weiteren Verbesserung des Curriculums begleitet werden.

## 1. Introduction

Statements such as “a 25% reduction of the risk through early identification of cancer”, “medicine X reduces the burden of symptoms 67%”, and “the severity of disease was significantly reduced by medicine Z” are often the basis of medical decisions, but they are difficult to comprehend, especially for lay people. Comprehensive informing of patients about the benefits and risks of examinations, therapies, or preventive measures is a prerequisite for shared decision-making (SDM), which has been increasingly promoted in recent years [5]. To do that, physicians need not only the ability to understand statistics themselves but also the ability to communicate them in a way patients can understand. This so-called communication of risk is an essential building block for sufficient implementation of SDM [6], [7], [8].

Yet most physicians lack the ability to communicate risk well. Many physicians themselves have difficulty with statistical values: they cannot distinguish relative from absolute risks, they equate higher five-year survival rates with lower mortality, and they cannot correctly specify the positive predictive value of tests [9], [10], [11], [12], [13], [14]. This inability to correctly grasp the meaning of statistical values is called “statistical illiteracy” and exists also in the general population and other health professions [9]. 

Furthermore, physicians’ evaluation of medical treatments is influenced by conflicts of interest, which arise from, among other sources, interactions of physicians with pharmaceutical companies [10], [11]. Statistical distortions and inappropriate presentations and interpretations of study data are consciously deployed, in order to let the data appear positive in the interests of the pharmaceutical companies. This distorted presentation of information in scientific publications and brochures from pharmaceutical companies further increases the difficulty of physicians to correctly evaluate the risks [12], [14], [15], resulting in an overestimation of the benefits and an underestimation of the risks of medical treatments [10], [11]. Most physicians are not conscious of their own susceptibility to influence, a phenomena that is called the “bias blind spot” [12], [13]. The “statistical illiteracy” described above leads therefore, in combination with the influence of conflicts of interests, to a systematic misinterpretation of scientific findings.

Despite the evidence for insufficient competence of physicians to communicate risks, neither the communication of risks nor the effects of and management of conflicts of interest have been comprehensively anchored in the curriculum so far [1], [2], [3], [4]. Nonetheless, the* National Competence-Based Catalog of Learning Goals in Medicine*, which was adopted in 2015, names these abilities in several learning goals. So the goal of the project described here was the development and implementation of an integrated model curriculum on the topics of communicating risk, bias, and conflicts of interests, as well as the evaluation of the feasibility of the curriculum and its acceptance by the students.

## 2. Project Description

### Approach

The development of the curriculum took place based on the 6-step cycle of Kern et al, which consists of the following steps: definition of the problem, needs assessment, definition of goals, selection of adequate methods, implementation, and evaluation [16]. This should not be understood as a linear process but instead as a continuous process, in which the methods can be adapted again and again through a renewed evaluation. The target group were medical students with basic knowledge of statistics and communication, i.e. in the 7^th^ to 10^th^ semester.

#### Definition of the Problem and Needs Assessment

After a literature search and based on the earlier work of the relevant workgroups [12], [15], [17], [18], physician’s lacking competency for communication of risk, described in the Introduction, was defined as the problem, which can be intensified through the influence of conflicts of interest, especially in interaction with industry. Both communication of risks and also conflicts of interest have not so far been comprehensively anchored in the curriculum. Currently, only individual aspects of communication of risks are taught in a fragmented way. The psychological aspects of general doctor-patient communication are taught and practiced in courses from the Medical Psychology departments during the preclinical years of the study program. During the clinical years of the study program, the students practice communication bedside. Statistical knowledge and evidence-based medicine are taught mostly by the departments of Statistics, dissociated from aspects of doctor patient communication. By contrast, conflicts of interest are only taught at all at a few universities [17], [19]. At the universities where conflicts of interest are taught, it is mostly placed in the domain of Ethics. Due to this division of individual learning topics among various subjects over the years of study, the integration of these topics into a unified picture does not occur for the students in the subsequent clinical routine. Fundamental concepts for good communication of risks and for the recognition of biases as well as management of conflicts of interest are not brought into relationship with each other by the students and therefore are not used in conversation with patients for adequate advising and decision-making.

Surveys of students have shown repeatedly that there is a need for more instruction about conflicts of interests [17], [20]. There is also a need for activity for the conveying of contents on communication of risk.Thus in a multiple-choice test of knowledge, taken in 2015 by over 1000 students of various medical schools, only 1 out of every 2 students could correctly answer the five questions on communication of risks (unpublished results). 

So the formal goal of this project was the development of a curriculum that can be adapted to different settings and thus can serve as a model, in order to make easier the time-efficient and resource-efficient introduction of corresponding courses at various universities. The content of the curriculum should combine basic knowledge from statistics with practice of communicating risk with patients, in order to assure the transfer of the learning content as well as the integration of the various topical areas. The overarching learning goal was defined as the acquisition of competency for communication of risk, i.e. after participating in the course, the students should be able to explain the benefits and risks of a therapeutic or screening intervention to patients in a comprehensible way, on the basis of statistics. The subordinate learning goals of the curriculum required for this were taken from the chapters, “Scholar”, “Professional Agent”, “Medical Scientific Skills”, and “Doctor-Patient Communication” of the *National Competence-Based Catalog of Learning Goals in Medicine* (see table 1 (Tab. 1)).

#### Formulation of Goals and Selection of Adequate Measures

A first draft of the curriculum, which included 16 lesson units (12 hours) was presented and discussed in the context of a 2-day expert workshop at the Harding Center for Risk Literacy. Among the 10 experts were teachers from the University medical schools of Berlin, Hamburg, and Aachen, who already teach various aspects of the topics of conflicts of interest and communication of risk at their medical schools and who were members of the Harding Center for Risk Literacy. The feedback of the experts led above all to a reduction of the themes. Thus, a thorough presentation of SDM was dropped, since this is already taught elsewhere. Also the management of conflicts of interest in research was shortened, in order to focus on the aspects relevant in the clinical domain. Nonetheless, the curriculum had to be expanded to 14.5 hours (19 lesson units), in order to integrate all the essential topics.

#### Implementation and Evaluation

The curriculum was first piloted in the summer semester 2016 with 13 students (8^th^ to 13^th^ semester) of the University of Heidelberg. Subsequently, the units on conflicts of interest among others were made more interactive in their methods and more differentiated in their contents, so there was more space for critical discussion. The revised version of the curriculum was carried out three more times at the universities of Mainz and Heidelberg in the framework of a study.

The evaluation of the pilot curriculum and the revised curriculum took place formatively in the form of oral and written feedback from the students and summatively in the form of grading of the curriculum by the students and a numerical evaluation of their own learning success and various aspects of the course. A self-evaluation questionnaire and a standard evaluation questionnaire were used for the summative evaluation (see attachment 1 ), [21].

## 3. Results

### Curriculum

The 14.5 hour (19 lesson units) curriculum integrates the fundamentals of statistics, theory and practical exercises on the communication of risk, the mechanisms of action of conflicts of interests, recognition of distortions in data, and possibilities for professional management of conflicts of interests, in order to avoid biases. Table 2 (Tab. 2) provides an overview.

#### Communication of Risk

As the basis for the communication of risks, statistical values were conveyed and simultaneously the direct relation to conversations with patients was established through concrete example formulations. These fundamentals are distributed among values that are relevant to the assessment of screening interventions and therapies. Using examples from published studies, the students calculate for example absolute and relative risks or risk reductions in experimental and control groups, e.g. for PSA screening [22]. The facts box – a form of health information that presents potential risks and benefits of medical procedures in a written form that is comprehensible for patients – is introduced as a method, and a fact box is made by the students themselves from a study [23], [24]. Essential components of good verbal communication of risks, in which the concept of SDM is embedded and discussed, are presented, such as the use of natural frequencies, in order to ensure the understanding of risk data by the patients. In order to make the transfers in their own conversations with patients easier, the students receive formulation aids, on how values can be communicated in comprehensible ways to patients.

#### Conflicts of Interests

Starting from the definition of conflicts of interests [25], the interests in the healthcare field that can run into conflict with each other are discussed. The focus of the curriculum is on conflicts of interests that can arise from interactions between pharmaceutical companies and clinically active physicians. Current data on the frequency and type of interaction between pharmaceutical companies and physicians or medical students are presented and discussed. The conflicts of interest resulting therefrom or the effects of the interactions on the clinical actions of the physicians is elucidated using example scenarios. The meaning of psychological mechanisms such as e.g. reciprocity or the bias blind spot are presented as an explanation for the influence of conflicts of interest and analyzed using example interactions. In order to illuminate how the representation of research results can be influenced by conflicts of interests, examples from brochures of the pharmaceutical industry are shown, such as e.g. presentation of relative risk instead of absolute risk or distorted graphs. For the constructive management of conflicts of interests, possibilities for the regulation of conflicts of interests in the areas of teaching, research, and patient care, as well as individual options for action are developed with the students.

#### Didactic Methods

For the choice of methodology, various approaches of learning theory were used. On the one hand, the principles of adult learning theory were important, in particular the assumptions that adults value it when what is being learned has a connection to the demands of their daily life (here: their occupational demands) and are more interested in problem-oriented approaches than in theme-based ones [26]. Furthermore, we integrated as many interactive lesson units as possible, corresponding to the hypothesis that interactive learning leads to greater learning success than constructive, active, or passive modes of learning (ICAP hypothesis) [27]. Finally, the concept of “deliberate practice”, as described by Ericsson, represented an important basis for the design of the exercise units with peers and simulation patients (SPs) [28].

The imparting of the necessary basics on the topics of conflicts of interests and communication of risks took place in lectures. Attention was given to activating the participants as often as possible through moderated discussions and open questions and thus to create opportunities to connect the contents with what was already learned. Moderated discussions were used, in order to make explicit the various perceptions of the participants and to reflect on them, above all in the thematic area of conflicts of interest. The statistical contents of the thematic area of risk communication were repeated and solidified in small group work. For the practice and solidification of communicational contents, the students carried out conversations in the context of role plays with each other and with SPs and received, besides their own video analysis of the conversation situation, feedback from SPs, peers, and instructors. The preparation of conversations, in which it was a matter of preference-sensitive decision situations, such as e.g. advising on cancer prevention examinations or the choice between two different treatment options for depression, was done individually. The students received information on the upcoming screening measures or various treatment options that was distorted graphically or content-wise yet from which they had to read out or infer statistics that were relevant for the upcoming case. Through group puzzles (changing group compositions, first with students who had done the same case, and subsequently in groups with students who had done other cases), the students subsequently compared experiences of the individual case scenarios. In subsequent 10-minute conversations with the SPs, the students ascertained their preferences and informed them accordingly about the benefits and risks of the procedures.

#### Evaluation

The curriculum was first piloted at the medical school of Heidelberg with a group of 13 students (mean (SD) age: 25.2 (2.4), 8/13 (61.5%) female, 8^th^-10^th^ semester) as an intensive weekend course (June 10-12, 2016).

The version of the curriculum that was revised according to the pilot findings was delivered three times in the same format in groups of 7-11 students in November and December 2017 at the Universities of Mainz and Heidelberg. The sociodemographic characteristics of the participants are presented in table 3 (Tab. 3).

The pilot of the curriculum was rated overall by the 13 students with a mean (SD) [range] grade of 1.5 (0.66) [1-3] on the 1-6 German school grading scale; the final curriculum was rated by 27 students with a mean (SD) [range] of 1.4 (0.49) [1-2]. In the evaluation questionnaire, the students rated 12 or 15 of 24 items on the general learning results or on the course with a mean above 5 points on a Likert scale from 1 (do not agree at all) to 6 (agree entirely), among others the items about methods competency, the structure of the course, the practical exercises, and the course atmosphere (see table 4 (Tab. 4) and table 5 (Tab. 5)). The requirements of the course were evaluated as suitable (mean (SD): 3.23 (0.44) for the pilot version and 3.00 (0.39) for the final version). They consistently rated their own competencies with a mean above 4 (see table 6 (Tab. 6)). These quantitative results were reflected in the open-text commentaries, which were clearly much more often positive than negative. Table 7 (Tab. 7) provides an impression through examples of the written commentary on the final curriculum. The oral feedback was comparable.

## 4. Discussion

The curriculum we developed plugs a gap in the current medical education. It arose in a structured development process that first identified positive examples of teaching on this topic in the literature. Where the literature was not fruitful, we used the input of experts in the framework of a workshop. The combination of theory and practice of the communication of risk, which until now have been taught separately, is innovative. This makes clear to the students the relevance of basic statistics, which otherwise are often evaluated as “dry”. Through clinically realistic exercises with the SPs, the transfer into the treatment context and the integration of the individual aspects is supported further. Imparting the fundamentals of the effect of conflicts of interest and their management makes the students able to recognize systematic distortions in data and to professionally manage conflicts of interest, in order to avoid biases in their own perceptions.

Already the pilot version of the curriculum was evaluated predominantly positively, as was the revised and improved version that was delivered three times in Heidelberg and Mainz. The long-term effect of the integrated curriculum was assessed in the framework of a randomized controlled study, which has been reported in detail elsewhere [29]. The students who took part in the piloting of the curriculum noticed, as we expected, the combination of theory and practice and also an interactive imparting of a dry subject. Moreover, they perceived the many feedback opportunities from various perspectives (SPs, teachers, peers) as very helpful. There were however points of criticism. In particular, they faulted the examples used for the role-plays as being too complex for the time available for them, which made it difficult to directly apply what they had learned. Furthermore, the density of information on the second day of the curriculum was criticized. Since we were aware of this, we tried to take into account the insights of cognitive load theory, in order to maximize the learning success of the students. Finally, many students remarked that the curriculum was too focused on the transmission of numerical information, which is perhaps not suitable for all patients. It is clear from this that the curriculum was accepted very well by the students, yet the organization of the contents can be further optimized, by adapting the role-plays and by additionally mentioning other communicational strategies besides the imparting of data for advising in the framework of SDM.

In the context of the BMG-supported project, “Pilot Implementation of the National Longitudinal Model Curriculum’s Communication in Medicine”, initially the medical schools of Hamburg, Heidelberg, Mainz, and Magdeburg are endeavoring to implement the curriculum into the teaching. There are various challenges to master for this. On the one hand, the cooperation of several subject disciplines is necessary for the introduction of the curriculum. Furthermore, the contents taught must be coordinated with the already established course. Finally, the training of the SPs requires some resources. At the medical school of Mainz, parts of the curriculum were integrated for the first time into the required coursework in the summer semester of 2018 and carried out with 186 students. The results of the evaluation were dispersed from grades of 1 to 4 (on a 1-6 scale), depending on the instructor. The biggest problem factor named by the students was the brevity of time: only 8 of the 19 teaching units were integrated in coordination with the already existing curriculum there. So for example, the statistics content was previously taught in the context of the course on evidence-based medicine (EBM), so these lessons from the curriculum we developed were not used. In their evaluations, the students recommended more intensive need for coordination between the various courses. During the second run in the winter semester of 2018-2019, the recommendations of the students were already taken into consideration, which led to a better coordination of the courses. The implementation of the curriculum for 200 students is demanding and costly and should be viewed as a continuous process of improvement in the sense of the Kern cycle.

Yet the investment seems entirely justified, in consideration of the positive feedback of the students as well as in consideration of the higher learning effect achieved, as reported elsewhere [29]. Besides the medical schools already named, where the first practical tests are carried out, several other medical schools have already expressed interest in wanting to establish the curriculum.

## 5. Conclusion

The model curriculum we developed on conflicts of interest and communication of risk was evaluated positively by the students. In the coming time, the practical introduction should be be tested at the four medical schools in the context of the longitudinal communication project. It would be desirable long-term to establish the curriculum at all medical schools in Germany and to improve it further in the framework of a continuous process on the basis of experiences in real practice.

## Funding

The project was financed by the Volkswagen Foundation (Reference Number 88574, to ND, JJ, and KL).

## Authors

These authors contributed equally to the publication: Nicole Deis, Cora Koch, Jana Jünger, Klaus Lieb. 

## Acknowledgements

We would like to thank Michael Hanna, PhD, (Mercury Medical Research & Writing) for translating the accepted final German version of the paper into English.

## Competing interests

The authors declare that they have no competing interests. 

## Supplementary Material

Evaluation questionnaire

## Figures and Tables

**Table 1:**
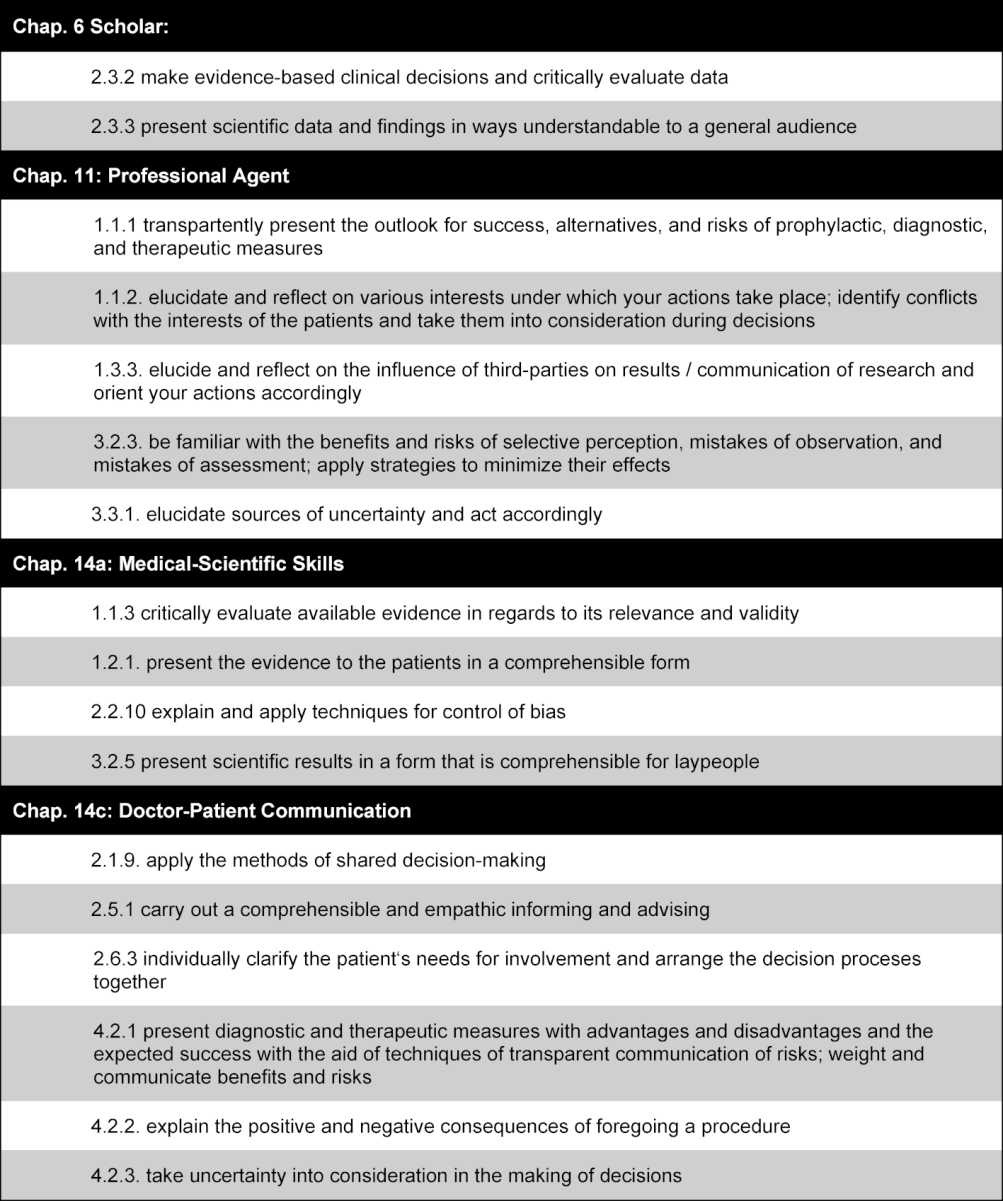
Learning Goals on Conflicts of Interest and Communication of Risk from the “National Competence-Based Catalog of Learning Goals in Medicine”

**Table 2:**
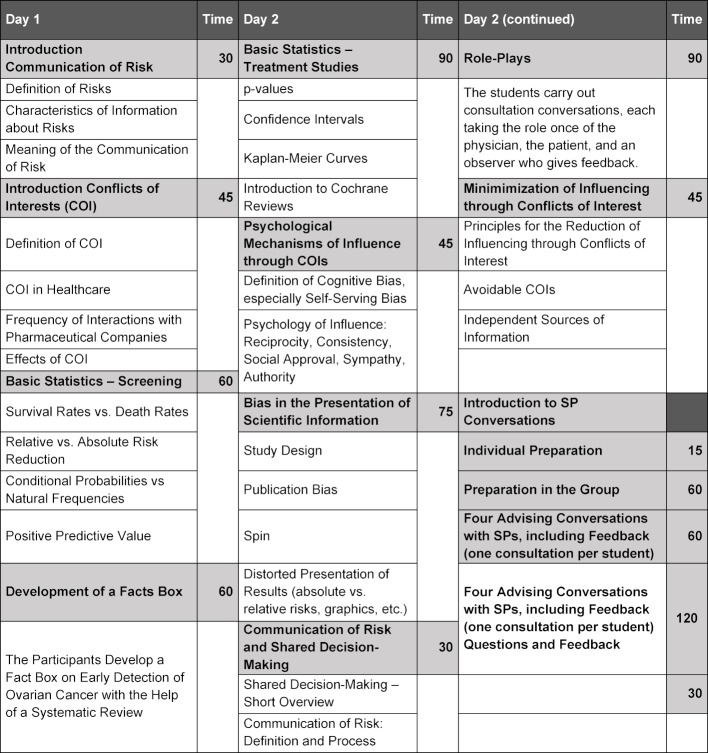
Overview of the Curriculum

**Table 3:**
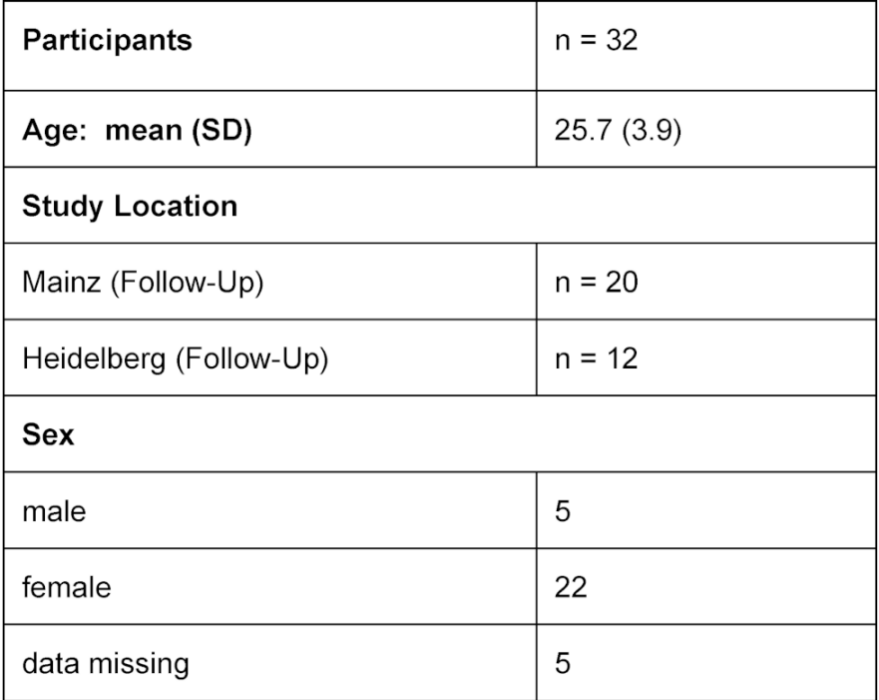
Sociodemographic Data

**Table 4:**
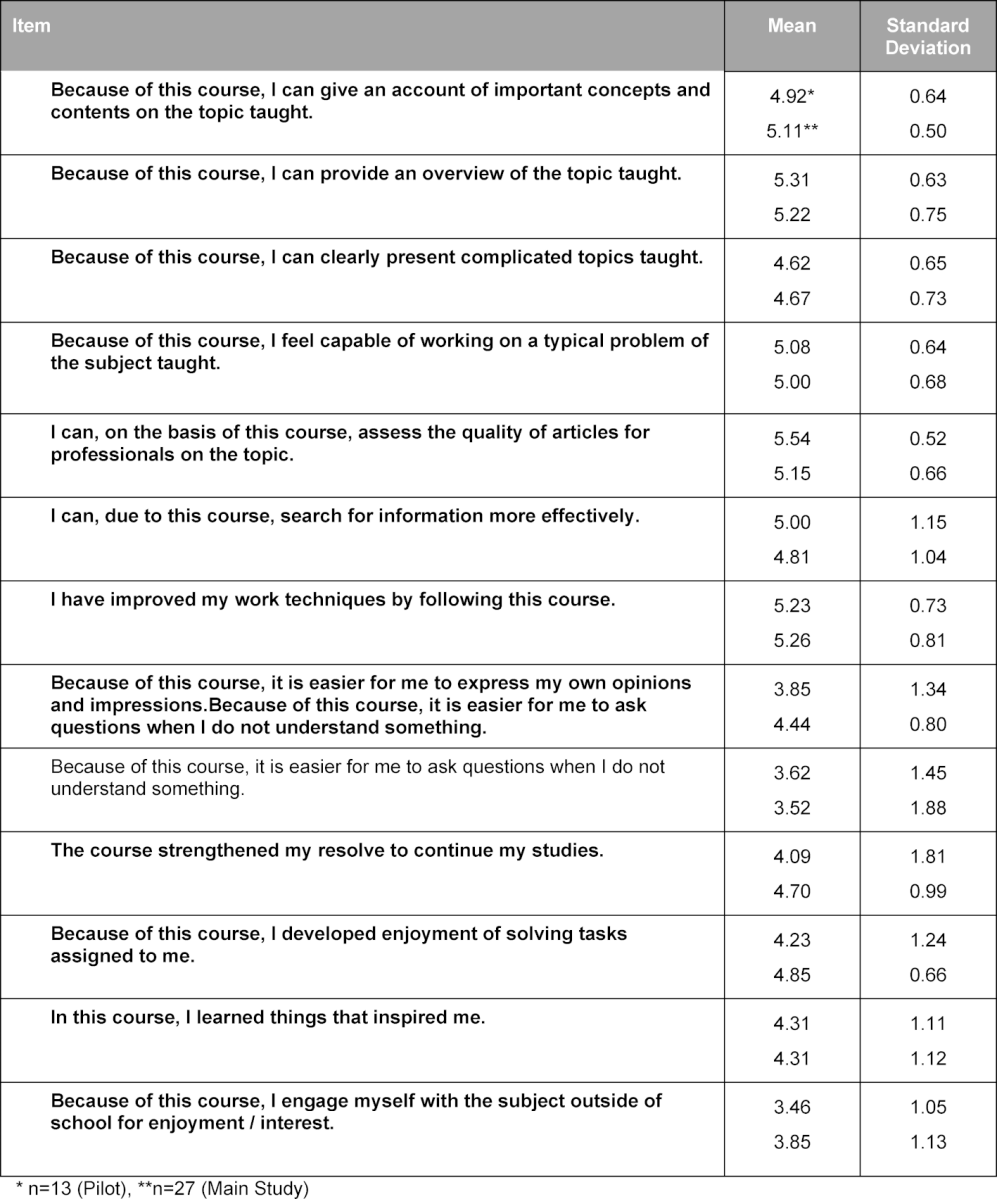
Self-Evaluation of the Students’ Learning Outcomes

**Table 5:**
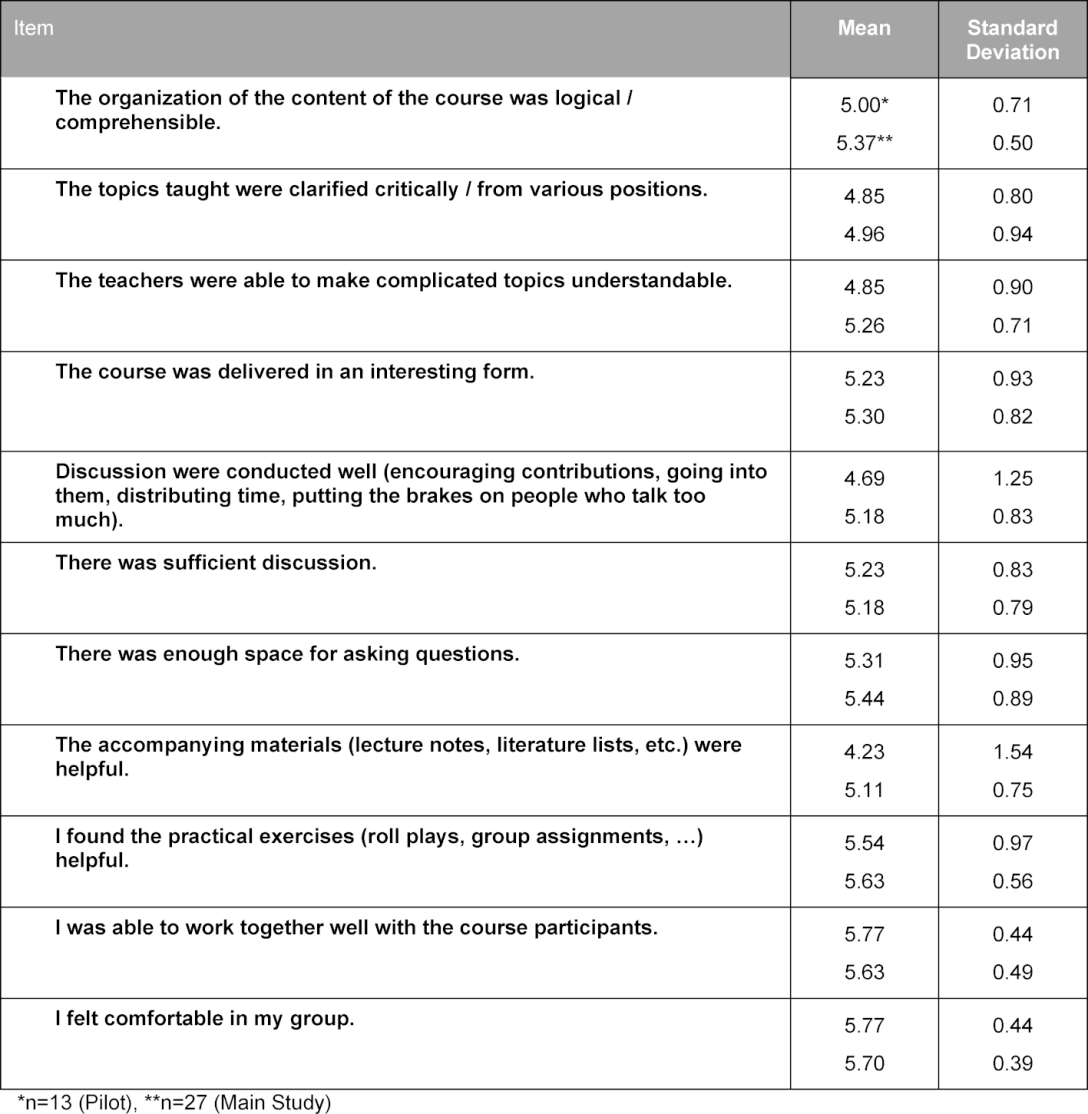
Evaluation of the Course

**Table 6:**
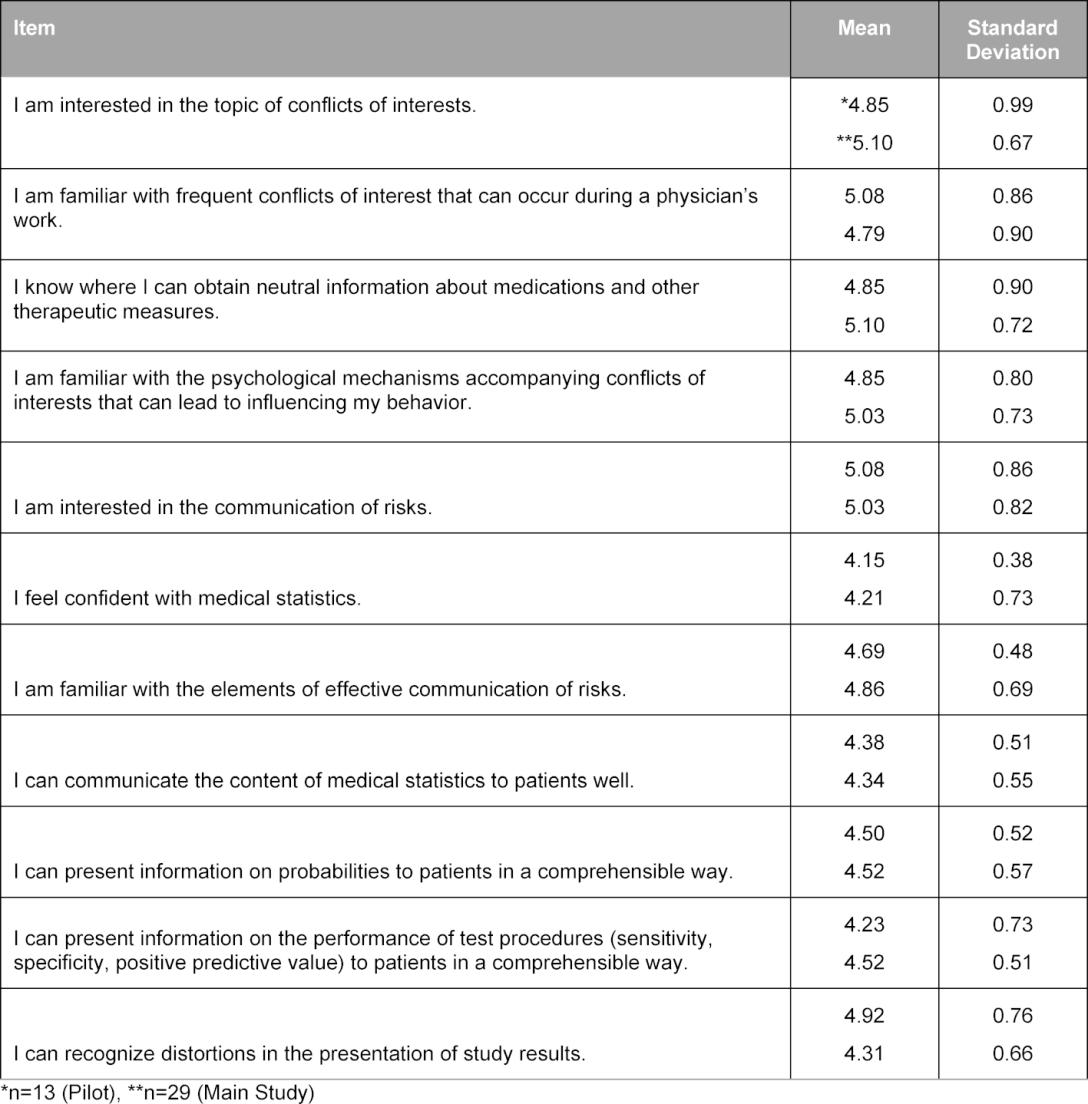
Self-Evaluation by the Students of Their Own Competencies

**Table 7:**
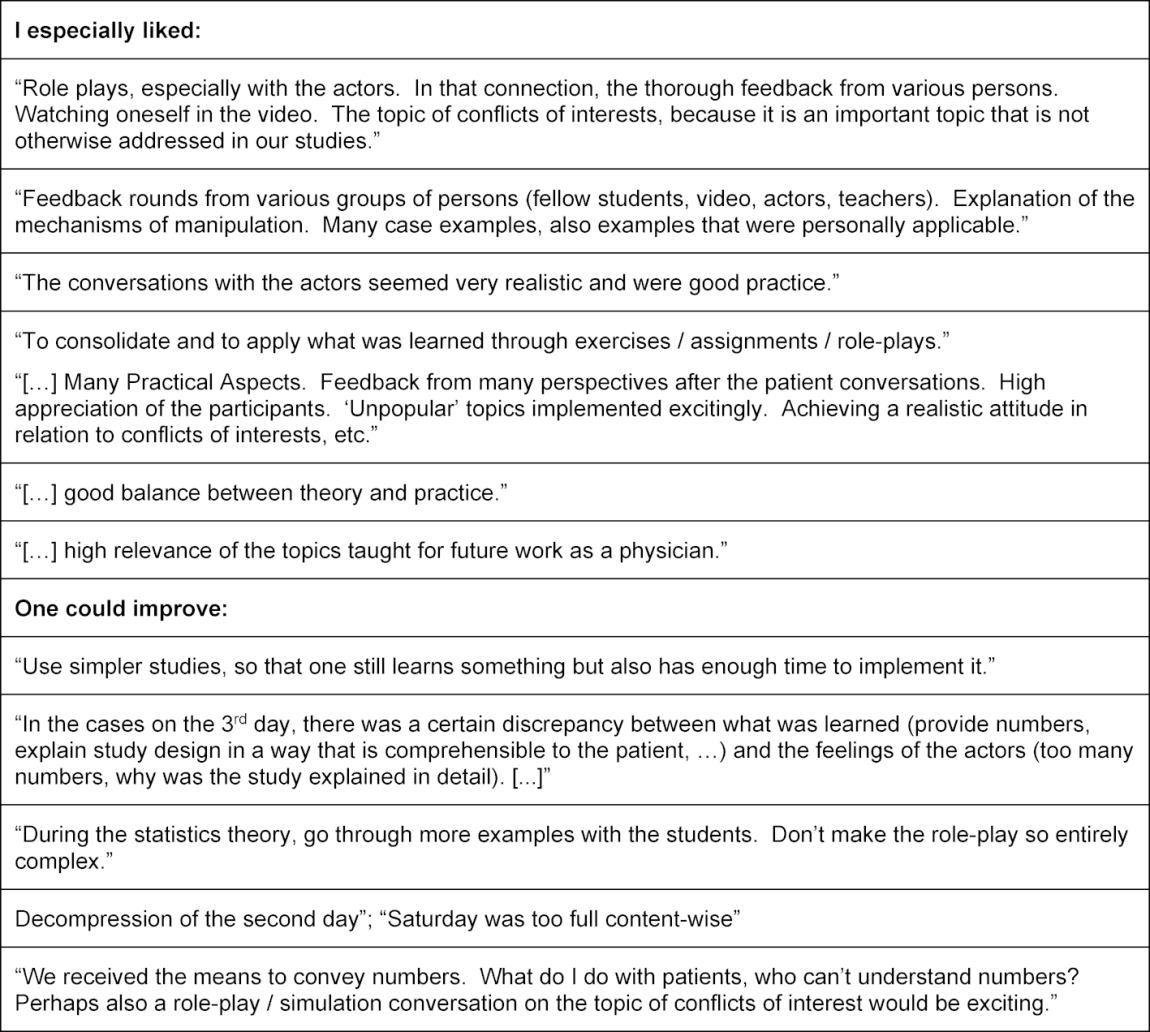
Open-Text Feedback
