# Supplementary material for: Development, implementation, and evaluation of a curriculum for medical students on conflicts of interest and communicating risk
Source: GMS J Med Educ. 2020 Feb 17;37(1):Doc3. doi: 10.3205/zma001296 (PMC7105765; doi:10.3205/zma001296)
Supplement: Evaluation questionnaire [file JME-37-3-s-001.pdf]

Evaluation

1. In the following, we ask you to evaluate only your learning results.

	not at all true	not true	somewhat not true	somewhat true	true	completely true
1. Because of this course, I can give an account of important concepts and contents on the topic taught.	☐	☐	☐	☐	☐	☐
2. Because of this course, I can provide an overview of the topic taught.	☐	☐	☐	☐	☐	☐
3. Because of this course, I can clearly present complicated topics taught.	☐	☐	☐	☐	☐	☐
4. Because of this course, I feel capable of working on a typical problem of the subject taught.	☐	☐	☐	☐	☐	☐
5. I can, on the basis of this course, assess the quality of articles for professionals on the topic.	☐	☐	☐	☐	☐	☐
6. I can, due to this course, search for information more effectively.	☐	☐	☐	☐	☐	☐
7. I have improved my work techniques by following this course.	☐	☐	☐	☐	☐	☐
8. Because of this course, it is easier for me to express my own opinions and impressions.	☐	☐	☐	☐	☐	☐
9. Because of this course, it is easier for me to	☐	☐	☐	☐	☐	☐

ask questions when I do not understand something.						
	not at all true	not true	somewhat not true	somewhat true	true	completely true
10. The course strengthened my resolve to continue my studies.	☐	☐	☐	☐	☐	☐
11. Because of this course, I developed enjoyment of solving tasks assigned to me.	☐	☐	☐	☐	☐	☐
12. In this course, I learned things that inspired me.	☐	☐	☐	☐	☐	☐
13. Because of this course, I engage myself with the subject outside of school for enjoyment / interest.	☐	☐	☐	☐	☐	☐

2. In the following, we ask you to evaluate only the seminar.

	not at all true	not true	somewhat not true	somewhat true	true	completely true
1. The organization of the content of the course was logical / comprehensible.	☐	☐	☐	☐	☐	☐
14. The topics taught were clarified critically / from various positions.	☐	☐	☐	☐	☐	☐
15. The teachers were able to make complicated topics understandable.	☐	☐	☐	☐	☐	☐
16. The course was delivered in an interesting form.	☐	☐	☐	☐	☐	☐
17. Discussion were conducted well (encouraging contributions, going into them, distributing time, putting the brakes on people who talk too much).	☐	☐	☐	☐	☐	☐
18. There was sufficient discussion.	☐	☐	☐	☐	☐	☐
19. There was enough space for asking questions.	☐	☐	☐	☐	☐	☐
20. The accompanying materials (lecture notes, literature lists, etc.) were helpful.	☐	☐	☐	☐	☐	☐
21. I found the practical exercises (roll plays, group assignments, …) helpful.	☐	☐	☐	☐	☐	☐
22. I was able to work together well with the course participants.	☐	☐	☐	☐	☐	☐
23. I felt comfortable in my group.	☐	☐	☐	☐	☐	☐

3. The demands in this course were …

… too low … too high

☐ ☐ ☐ ☐ ☐

4. **How do you evaluate the efforts required before and after the course in relation to the learning goals?**

   … too low … too high

   ☐ ☐ ☐ ☐ ☐

5. **How many sessions of the course did you attend?**

the first		the second		the third	
☐yes	☐no	☐yes	☐no	☐yes	☐no

6. **How much time did you spend preparing for this course and reviewing it afterwards?**

   ________ hours

7. **I give the course the following school grade overall:**

1	2	3	4	5	6
☐	☐	☐	☐	☐	☐

8. I especially liked:

9. One could improve:
